# Characteristics of an Effective International Humanitarian Assistance: A Systematic Review

**DOI:** 10.1371/currents.dis.706b7dc0e8382b55a20d6f7d0cf14257

**Published:** 2016-02-25

**Authors:** Shandiz Moslehi, Ali Ardalan, William Waugh, Daniel C. Tirone, Ali Akbarisari

**Affiliations:** Department of Disaster Public Health, School of public Health, Tehran University of Medical Sciences, Tehran, Iran; Department of Disaster and Emergency Health, National Institute of Health Research, Tehran University of Medical Sciences, Tehran, Iran; Disaster and Emergency Health Academy, National Institute of Health Research, School of Public Health, Tehran University of Medical Sciences, Tehran, Iran; Harvard Humanitarian Initiative, Harvard University, Cambridge, USA; Department of Public Management and Policy, Andrew Young School of Policy Studies, Georgia State University, Georgia, USA; Department of Political Science, Louisiana State University, Baton Rouge, Louisiana, USA; Department of Health Care Management, School of Public Health, Tehran University of Medical Sciences, Tehran, Iran

**Keywords:** effectiveness, humanitarian aid, humanitarian assistance, relief

## Abstract

Introduction: The objective of this study is to identify the effectiveness characteristics, review the definition of them, and develop a conceptual mapping of existing domains in the field of International Humanitarian Assistance (IHA).

Methods: We conducted a systematic review and searched the major databases (Science Direct, Scopus, Springer and Pubmed) and grey literature, including references of potentially eligible articles and conference proceedings through March 2015. Articles were included if they focused on IHA effectiveness. Reviewers independently identified the eligible studies and extracted data.

Results: 10 studies were included and 48 characteristics were identified. There is a lack of scientific studies and agreement on the characteristics of IHA effectiveness.

Conclusion: This study could be the step toward an understanding of IHA effectiveness characteristics and its definitions with the findings making a base line for more research in this area.

## Introduction

According to the trends in the data, the number of reported disasters and the numbers of people killed or otherwise affected by natural disasters have increased over the past 50 years 1. Based on the Office for the Coordination of Humanitarian Affairs (OCHA) report of global humanitarian response 2014, 17 countries were affected; 568 aid organizations participated in Humanitarian Assistance (HA); and there was a large gap between funded and requested fund programs^1^^,^ 2. It is estimated that 102 million people are in need of HA, while it was 81 million in December 2013. Global financial requirements to cover humanitarian needs are US$17.3 billion compared with US$12.9 billion in 2013^3^. Such increases in the level of HA funds are leading to the increased importance of effective allocation^4^^,^ 5.

In 1962, Morgenthou defined the HA as a foreign aid which governments extend to nations faced with natural disasters^6^. In this definition the author did not mention the man-made disasters such as wars and armed conflicts. OCHA defines the HA as: “Aid that seeks, to save lives and alleviate suffering of a crisis-affected population” 7. The aim of HA is to protect the lives of the affected people during and after disasters^8^. International HA (IHA) will be provided when the governments of the affected countries are unable to provide relief in an effective way. The assistance is provided in emergency situations to save and protect human life in both natural and man-made disasters and to maintain human dignity^9^. This assistance should be allocated in an effective way in order to maximize its impact.

Etzioni in 1964 was one of the first researchers who defined the goal attainment approach of effectiveness^10^^,^ 11. Georgopoulos and Tannenbaum in 1957 and Yuchtman and Seashore in 1967 emphasized a financial approach 12 ^,^ 13. The approach of reputation was evolved from Georgopoulos and Mann’s project in the hospital system^14^. This approach relies on subjective performance measures reported by informants or organizational stakeholders^15^. After that the literature focused on the definition of more complex methods of effectiveness, such as multi-dimension models 16 ^,^ 17 ^,^ 18, competing values models 19, contingency models 20, and balanced scorecard^21^ approaches. These methods tried to unify the aspects of goal attainment, resource control and reputation approaches 22.

HA effectiveness is defined as: objectives achievement^23^ and the extent to which the interventions’ immediate objectives were achieved, measured by the timeliness of payments and the targeting of aid on the needs of affected individuals^24^. As recognized at the Consultation Workshop for Humanitarian Effectiveness in 2013, identifying ways of defining, measuring and improving the effectiveness of HA was one of the thematic areas of focus for the World Humanitarian Summit (WHS)^25^. Defining clear targets for improving aid effectiveness was the result of Paris Conference on Aid Effectiveness in March 2005 26 . During the Rome Conference on Harmonization and Alignment in 2003, about 50 countries spent two days discussing the ways they could improve the effectiveness of their works^26^. Also at the Monterrey Conference on Finance for Development in 2002, it was mentioned that increasing aid volume will not reduce the poverty. Instead, the aid should be more effective^27^.

IHA organizations are expected to save lives with limited resources^28^. Measuring the effectiveness of IHA became an OCHA priority in 2013-2014^25^, increasing humanitarian actions more than 6 fold from 1990 to 2012^29^. The shortage of empirical studies about effectiveness of IHA lead us to conduct this study. So the objective of this study is to identify the effectiveness characteristics, review their definitions and develop a conceptual mapping of existing characteristics in the field of IHA.

## Methods

**Study design**

The approach is comprehensive and involves a search strategy including international, relevant studies. It followed the Preferred Reporting Items for Systematic Review and Meta-analysis statement (PRISMA) guidelines. In addition, we reviewed the references in potentially eligible articles, conference proceedings, dissertations, organizational reports and documents of the major IHA organizations to minimize the publication bias.

**Search methods for identification of studies**

Search scientific articles:

We conducted a systematic search in electronic databases (from their inception to the latest available entry date) on 3 March 2015:including Science Direct, Scopus, Springer and Pubmed.

The search language was restricted to English. The search keywords were selected after consulting the experts. Databases were searched with the following keywords: humanitarian* AND assistance* OR relief* OR aid* AND effectiveness*. In addition, the references of relevant articles were reviewed. This helped to evaluate the effectiveness of the search strategy and to identify any articles overlooked due to unusual keywords.

Search other resources:

This search was conducted following organizational reports and documents: OXFAM, OCHA, Save the Children, Swedish International Development Cooperation Agency (SIDA), Canadian International Development Agency (CIDA), Active Learning Network for Accountability and Performance (ALNAP), Department for International Development (DFID) and Humanitarian Practice Network (HPN).

**Study eligibility**

Inclusion criteria:

Eligible studies included English-language articles with no limitation in time of studies. No other filters were applied. Articles were included if they focused on IHA and effectiveness characteristics. Both quantitative and qualitative studies were included. Also, organizational reports and documents were included.

Exclusion Criteria:

Non English studies, the irrelevant studies to IHA effectiveness and articles with similar keywords but covering unrelated topics were excluded.

**Data collection and analysis**

Selection of studies:

After excluding duplicated studies, references found through the search were evaluated for inclusion criteria by reading their titles and abstracts. After an initial screening of abstracts of those that remained, the first author (SM) read and analyzed their full texts to determine their eligibility. This was done under the supervision of second author (AA). The first author had passed IHA course at the Harvard Humanitarian Initiative. Quality assessment was performed using study design appropriate Critical Appraisal Skills Program (CASP) tools. Disagreements were resolved by consensus or by consulting other authors.

Data extraction and analysis:

We extracted the following data from each study report: organization; characteristics or elements influence the IHA effectiveness; and the definition of characteristics. Data extraction forms were designed using Microsoft Excel. Extracted data were presented in descriptive tables.

## Results

A total of 494,675 potentially relevant articles were identified during the search. The analysis contained 92 articles that met the inclusion criteria. A full text review of these articles led to 10 studies. The study selection flow diagram is shown in Figure 1 and Figure 2 shows the Description and Mapping of IHA effectiveness characteristics.

**The flow diagram for selection of studies:**
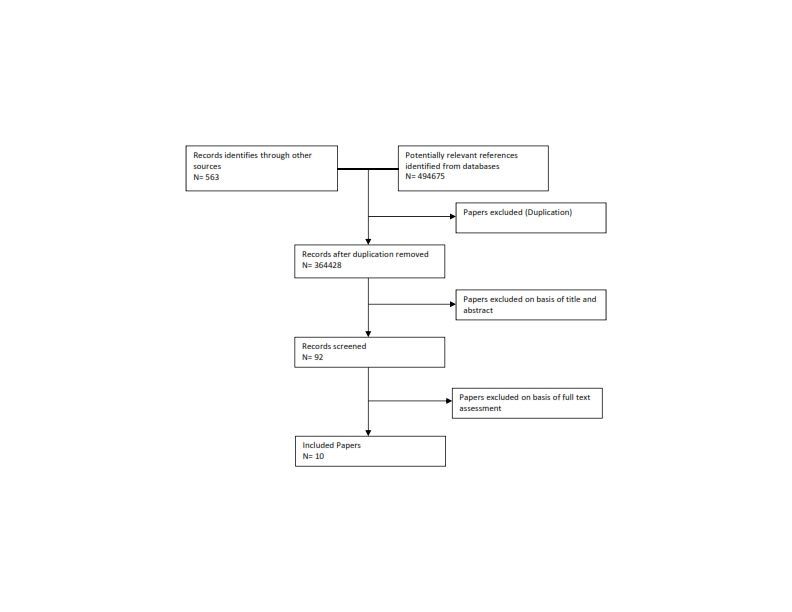

**Description and mapping of IHA effectiveness characteristics:**
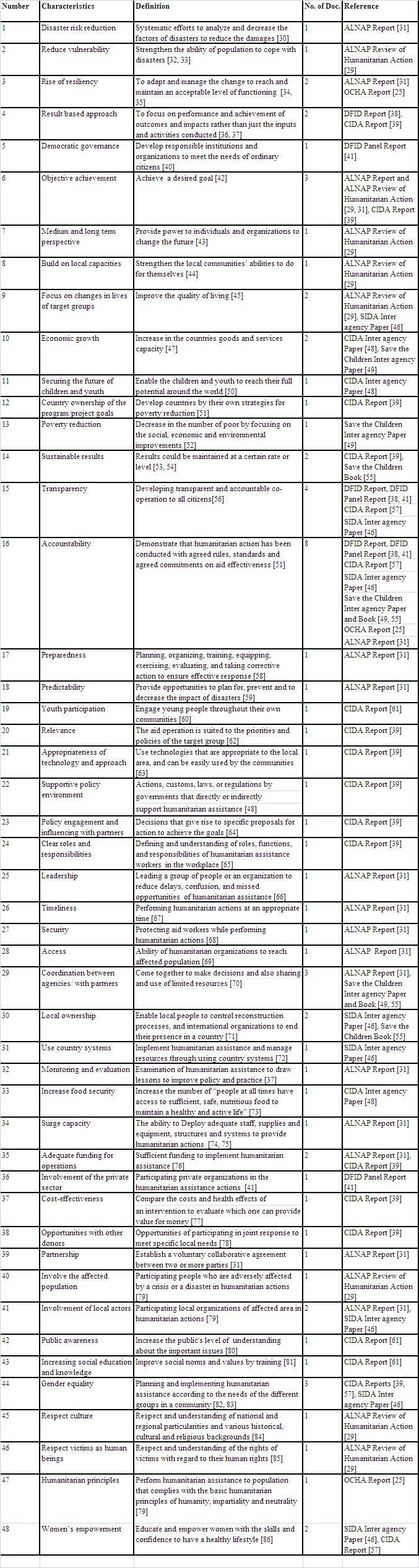

## Discussion

Based on our results, we could find the main characteristics of IHA effectiveness, but there was no standard definition for each of them. We tried to develop a conceptual mapping of existing characteristics of IHA effectiveness.

There is a matter of terminology in IHA subject and relief programs^28^. Although there were a lot of meetings for identifying IHA effectiveness 25 ^,^ 26, there is no single and accurate definition of IHA effectiveness 27. Organizations tend to report the positive aspects and ignore the negative points. Some may report the international efforts and the others may report the outcomes to define their effectiveness^87^. Governmental, intergovernmental, NGOs and other organizations may define the effectiveness due to their own perspectives 88. There is a global agreement on defining criteria which should be used to measure the IHA effectiveness. These criteria should help the decision makers to plan, implement and evaluate the IHA in a standard way 28. So, OCHA held a consultative workshop in 21 March 2013 to identify ways of defining, evaluating and improving IHA effectiveness 25.

We categorized and present the data found from IHA organizations documents and reports (the grey literature). The study results appear to be over-predicted and the number of studies found was lower than the value we expected. This confirms previous findings in the literature. Some studies agreed that HA evaluators face with the lack of appropriate data^28^. Also, all the humanitarian organizations do not upload their evaluation reports and documents on their websites to disseminate the results with other organizations or researchers 87 . Lack of access to reports and documents from IHA organizations can reduce the quality of aid provided 89 ^,^ 90.

The most striking result to emerge from the data was the lack of systematic reviews in the subject of HA. There is a global need for conducting HA systematic reviews. Because conducting systematic reviews could play an important role in increasing the effectiveness of IHA in all the phases of a disaster^91^. As mentioned by Bonnix Kayabu and Mike Clarke 83% of their study participants said that systematic reviews are useful in disasters and 69% of their participants strongly agreed that findings of systematic reviews with the subject of IHA could have a positive role in humanitarian programs^91^.

There was no standard category for these characteristics. In the Paris Declaration there were five defined categories: mutual accountability, managing for results, harmonization, alignment and ownership 51. In OECD Development Assistance Committee (OECD-DAC) there were also five defined categories: relevance, appropriateness, connectedness, efficiency, effectiveness and impact 25. This model was criticized because of being too developmentally focused and the absence of effectiveness indicators 25. We could not categorize all these characteristics in these defined categories.

**Limitations:**

The most important limitation of this study lies in the fact that we only used English articles, documents and reports. We may have missed some important published articles, documents and reports in other languages. The findings also might not be representative of all IHA organizations, since we had analyzed the documents of major IHA organizations.

**Conclusion:**

In this review we have presented the IHA effectiveness characteristics from previous reports and documents. The evidence from this study supports the idea that there is a lack of scientific studies in the field of IHA effectiveness. Also the findings of this study indicate that there is no agreed definition for IHA effectiveness characteristics, although there is a global need for conducting IHA effectiveness evaluation. We developed a conceptual mapping of existing characteristics of IHA effectiveness. Although this study is a first step toward our understanding of IHA effectiveness, the findings add to growing body of literature on IHA effectiveness.

## Supporting Information

PRISMA Checklist

## Competing Interest

The authors have declared that no competing interests exist.

## Correspondence

Ali Ardalan. Email: aardalan@tums.ac.ir, ardalan@hsph.harvard.edu

This article is a part of a PhD dissertation from Tehran University of Medical Sciences, Tehran, Iran.
